# Transforming Children’s Attitudes Toward Insects Through In-School Encounters

**DOI:** 10.3390/insects16010093

**Published:** 2025-01-17

**Authors:** Kathleen M. Miller, Dana K. Beegle, Stephanie Blevins Wycoff, Daniel L. Frank

**Affiliations:** Department of Entomology, College of Agriculture and Life Sciences, Virginia Tech, Blacksburg, VA 24061, USA; millerkm@vt.edu (K.M.M.); dbeegle@vt.edu (D.K.B.); slblevin@vt.edu (S.B.W.)

**Keywords:** entomology outreach events, elementary education, science literacy event, arthropods

## Abstract

Each year, the Department of Entomology at Virginia Tech hosts an entomology-themed outreach event known as Hokie BugFest. This on-campus, festival-sized experience aims to educate the public about insects and other arthropods through hands-on activities, games, displays, and live arthropods. In 2021, due to the COVID-19 pandemic, Hokie BugFest and similar large public events were cancelled. In response, the department launched Hokie BugFest on the Go, which offered smaller-scale, in-person learning opportunities during these closures. Instead of hosting the community on campus, Virginia Tech’s Department of Entomology brought live arthropods, university experts, and the exciting science of entomology directly into schools, fostering small-group, hands-on learning experiences. In 2022, a playful assessment was added to the traveling outreach program to measure changes in student attitudes and perceptions of insects and other arthropods before and after the program. The assessment also gauged students’ favorite arthropods after seeing, and in some cases handling, them live during the program. Assessment results revealed valuable insights into how hands-on, applied learning experiences can shift children’s attitudes toward arthropods. Results showed that even after expressing trepidation and fears, students’ knowledge and comfort levels with insects and other arthropods increased as they interacted and learned throughout the program. These findings underscore the value of using engaging, hands-on, small-group approaches when designing entomology-themed outreach events for young audiences and offer guidance for future programs.

## Simple Summary

The entomology-themed outreach event Hokie BugFest is held each year by the Department of Entomology at Virginia Tech. This large, one-day festival aims to educate the public about insects and other arthropods while promoting a greater appreciation of and interest in the topic. During the COVID-19 pandemic, Hokie BugFest and many other large, in-person outreach events closed. In response, Hokie BugFest on the Go was created in 2021 to provide in-person learning opportunities for students during those event closures. In 2022, a playful assessment was added to the program to gauge changes in students’ attitudes toward arthropods after they experienced the program. The results showed that even after expressing their fears, students’ comfort levels with insects and other arthropods increased as they interacted and learned. Understanding how fun, hands-on programs influence children’s attitudes can inform the design and development of similar entomology outreach events in the future.

## 1. Introduction

It is well-documented that insects and other arthropods play a vital role in our ecosystem; however, these animals are often misunderstood and undervalued by the general public [1,2,3]. In an effort to educate communities, numerous insect-focused literacy events, led by universities and other organizations, have been created both in the United States and internationally to further promote a greater appreciation and understanding of these diverse taxa [4,5,6]. However, despite the ecological significance of insects and the growing popularity of entomology-themed outreach, studies show that the public often still holds negative perceptions of and attitudes toward invertebrates, with insects frequently eliciting a reaction of disgust or fear [7,8,9].

While humans have an innate evolutionary aversion to insects [10], widespread disgust and fear of insects are likely reinforced by urbanization. Living in urban environments allows for a greater likelihood of seeing insects indoors, while also minimizing the ability of someone to experience them in nature. This prevents humans from developing an ability to distinguish between various arthropod species and their ecological role [10], such as whether an insect is a harmful pest or primarily beneficial. Educational programs using invertebrates have been found to effectively reduce associated disgust [7,11].

Due to their small size and ease of transport, insects and other arthropods are ideal tools for science education. These characteristics allow for a hands-on, up-close approach to learning about environmental and biological concepts [1,7]. However, in schools, teachers’ attitudes toward arthropods can influence their willingness to incorporate arthropod-focused lessons into the curriculum [8]. Teachers with positive attitudes regarding an animal are generally more inclined to include them in their lessons [12]. This, in turn, can influence students’ exposure to biodiverse concepts, affecting their attitudes toward, future engagement with, and overall understanding of arthropod-focused materials.

Each fall, the Department of Entomology at Virginia Tech hosts Hokie BugFest, a one-day event designed to generate interest in and awareness of entomology through hands-on science learning. Bringing in over 8000 guests annually, this event hosts over 35 exhibitor booths, offering attendees the opportunity to interact with live animals and university personnel, view pinned specimens, and participate in a scavenger hunt to earn a Jr. Entomologist Certificate. However, the onset of the COVID-19 pandemic in 2020 made large, in-person outreach events impossible. In response, Hokie BugFest transitioned to an online format, presenting a collection of asynchronous exhibitor booths that provided various educational resources, such as fun facts publications, videos, photo galleries, and downloadable activities.

Despite the success of the online Hokie BugFest, the programming lacked opportunities for direct interactions and discovery with live arthropods. To compensate, an additional initiative, Hokie BugFest on the Go, was developed in 2021 to continue delivering in-person learning opportunities to the area’s youth. Instead of the community coming to the university, Virginia Tech’s Department of Entomology brought live arthropods, university experts, and the exciting science of entomology directly to the schools. Students learned in small groups, fostering individualized dialogue and hands-on opportunities for each student. Between 2021 and 2022, the Hokie BugFest on the Go program reached hundreds of elementary-aged students in four school districts who may have been unable to attend an in-person Hokie BugFest. The traveling program also provided outside learning activities for schools with limited field trip budgets.

Here, we discuss the results of a playful assessment aimed at evaluating how students’ attitudes and perceptions of insects and other arthropods changed following their participation in the Hokie BugFest on the Go program. Furthermore, we offer insights and information to guide the design of effective entomology outreach events for future initiatives.

## 2. Materials and Methods

Volunteers from the Department of Entomology at Virginia Tech (faculty, staff, and graduate students) brought live arthropods and other educational resources to 11 and 6 public elementary schools in September 2021 and 2022, respectively. Within each school, learning stations were set up in a central space (e.g., gymnasium, library, cafeteria). Up to two classes at a time (averaging 31 students in total; predominately 3rd and 4th graders aged 8–10 years old) visited these central locations for a 1 h interactive learning session. Every session began with a 15 min presentation that introduced general entomology concepts. This included a basic description of arthropod and insect characteristics; the terms molting, metamorphosis, habitat, adaptation, and ecosystem; the role insects and arthropods play in our ecosystem; and entomology-focused careers. Following the opening presentation, students were divided into smaller groups (averaging 10 students per group) and rotated through three stations where they learned about a variety of live arthropods from Virginia Tech’s Bug Zoo. Each session featured at least the following arthropods: darkling beetles (*Asbolus verrucosus*), Madagascar hissing cockroaches (*Gromphadorhina portentosa*), mantids (*Stagmomantis carolina*), millipedes (Anadenobolus monilicornis), scorpions (*Hadrurus arizonensis*), tarantulas (*Grammostola rosea*), and termites (*Zootermopsis angusticollis*). The stations offered touch and hold opportunities and further engagement with university experts who highlighted unique characteristics of each arthropod. These included demonstrations of the darkling beetle’s death feigning abilities, hissing behavior of the Madagascar hissing cockroaches, and the mantid’s camouflage skills. Volunteers also showcased millipedes in leaf detritus and termites within a cross section of wood to better explain their roles as detritivores, illuminated scorpions under black light to reveal their fluorescent capabilities, and compared a tarantula’s molt with a live specimen to describe the molting process and provide a close-up view of its chelicerae, fangs, and hairs. At every station, non-exhaustive fact sheets were provided for each arthropod, including details about their habitat, diet, life span, distribution, and a fun fact. These fact sheets were written at a third- to fourth-grade reading level to ensure accessibility. After visiting each station, students came back together into one group to review the fundamental concepts covered at each station. Each concept discussed and vocabulary term learned were directly linked to Virginia Standards of Learning (SOLs).

To continue students’ excitement after the program and reinforce vocabulary and concepts learned, every student received a “My Hokie BugFest on the Go Field Trip” activity booklet (see Supplementary File S1), along with entomology swag (stickers, buttons, etc.) and an invitation to learn more online at the Hokie BugFest website (https://www.ento.vt.edu/4-H_Entomology/hokiebugfest.html, accessed 10 January 2025). Teachers also received an invitation to incorporate online Hokie BugFest content into their lesson plans, supported by an Educators’ Guide specifically developed for K-12 educators. The Educators’ Guide outlined the online exhibits from the Hokie BugFest website, categorizing them by content type (i.e., exhibit information and photo gallery, fun facts and publications, videos, or activities), grade/age suitability, relevant learning topic(s) or subject(s), and the Virginia SOLs addressed for each exhibit. This helped educators quickly and easily connect the content to their curriculum.

In 2022, a playful assessment was used to measure changes in students’ attitudes and perceptions of insects before and after the program. The Virginia Tech Institutional Review Board granted an exemption for data collection (VT IRB no. 22-732). Assessment activities included voting boxes, group question/answer, and an interactive leaderboard. While entering the learning space, each student received two strips of paper to cast their votes for completing the statement “Insects are …”. Their options for completing the statement were “cute” or “gross” and “fun” or “scary”. After voting, students were asked an additional series of yes or no questions that included the following: “Do you like insects?”, “Do you think insects are important to humans?”, and “Will you touch or hold an insect during the program?”. Students responded to questions by raising their hands, and responses were recorded by at least three university personnel located at the back of the space. These assessments were again repeated at the end of the session before students left the space, including “Did you touch or hold an insect during the program?”. Additionally, before the end of each session, students were asked two further questions. First, they responded to the yes or no question, “Will you continue learning about insects after the program?”. Second, students were invited to choose their favorite arthropod from the live specimens featured during the program. They did this by placing a sticker next to their selected arthropod on a leaderboard. Each group’s preferences were revealed in real time as the leaderboard chart was updated with each student’s vote.

The comparison of pre- and post-session assessment responses was performed using McNemar’s chi-square test (SAS 9.2, SAS Institute, Cary, NC, USA). Results from all tests were considered significantly different at *p* < 0.05.

## 3. Results

During 2021–2022, Hokie BugFest on the Go reached 1570 students across four public school districts in Southwest Virginia. Within these districts, the program engaged 87 classes at 17 elementary schools. By traveling directly to the schools, the program eliminated the need for schools to allocate funds for field trips, saving a total of USD 5520 in fees.

In 2022, 713 students participated in pre- and post-session assessments to evaluate their views on insects and other arthropods. Overall, results showed significant shifts in student attitudes and perceptions before versus after the program. Specifically, views of insects as “gross” versus “cute” changed significantly (*χ*^2^ = 101.83, df = 1, *p* < 0.0001). The percentage of students who indicated insects as “gross” decreased from 31.0% before the program to 15.4% after the program, whereas the percentage who indicated them as “cute” increased from 69.0% before the program to 84.6% after the program (Table 1). Similarly, views of insects as “scary” versus “fun” also changed significantly (*χ*^2^ = 92.35, df = 1, *p* < 0.0001). The percentage of students who indicated insects as “scary” decreased from 25.5% before the program to 11.8% after the program, whereas the percentage who indicated them as “fun” increased from 74.5% before the program to 88.2% after the program (Table 1).

Significant changes were observed in students’ responses to liking insects (*χ*^2^ = 38.00, df = 1, *p* < 0.0001). The percentage of students who indicated that they disliked insects decreased from 13.7% before the program to 8.4% after the program, whereas the percentage of students who indicated that they liked insects increased from 86.3% before the program to 91.6% after the program (Table 2). Similarly, significant changes were observed in students’ perception of insects as important to humans (*χ*^2^ = 19.00, df = 1, *p* < 0.0001). The percentage of students who indicated that insects were not important to humans decreased from 6.3% before the program to 3.6% after the program, whereas the percentage of students who indicated that insects were important to humans increased from 93.7% before the program to 96.4% after the program (Table 2). Additionally, significant changes were observed in students’ willingness to touch or hold an insect during the program (*χ*^2^ = 7.53, df = 1, *p* = 0.0061). The percentage of students who indicated they would not touch or hold an insect decreased from 10.0% before the program to 7.7% after the program, whereas the percentage of students who indicated that they would touch or hold an insect increased from 90.0% before the program to 92.3% after the program (Table 2).

**Figure 1:**
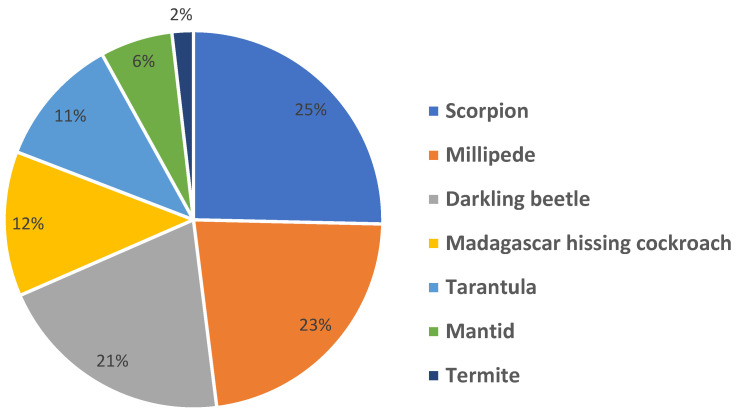
Leaderboard activity: students’ favorite arthropod from the program (*n* = 713).

Following the program, 94% of students indicated that they wanted to continue learning more about insects and other arthropods. Furthermore, among the live arthropods introduced during the program, scorpions were the most frequently chosen as students’ favorites, followed by millipedes, darkling beetles, Madagascar hissing cockroaches, tarantulas, mantids, and termites (Figure 1).

## 4. Discussion

Overall, the results showed that despite any initial fears some may have had, students were interested in learning about insects and arthropods. The results also revealed that students’ comfort levels increased as they learned and that students planned to continue learning about insects and arthropods after the program. Other studies have similarly shown an increase in comfort levels and a reduction in fears as participants learn, along with a desire by participants to continue learning about the subject matter in the future [6,13]. These findings also align with previous research showing that positive experiences with animals known to previously elicit fear can shift children’s attitudes and reduce feelings of disgust [7,8,14,15].

Notably, there was a significant shift in students’ overall attitudes toward insects and other arthropods, with descriptors like “gross” versus “cute” and “scary” versus “fun” changing positively after engaging with and learning about arthropods during the program. The significant increase in students liking insects and recognizing their importance to humans further supports previous findings that knowledge about insects and the environment, combined with positive interactions, can foster greater appreciation for these animals and their ecological roles [2,11]. Such appreciation and continued learning about entomology could ultimately contribute to conservation efforts.

During Hokie BugFest on the Go, program leaders identified several impactful strategies for working with children and arthropods. Understanding the elementary-aged audience is essential for effectively conveying entomological information in a fun and engaging manner. Starting with a playful assessment and informational session helped acclimate students to the topic of entomology, allowing them to become comfortable with the subject before engaging in hands-on activities and experiences. Following the informational session, students rotated among three stations, each featuring different arthropods and led by one to two university volunteers. Dividing students into smaller groups for these rotations provided more opportunities for one-on-one interactions with volunteers, allowing students to hold arthropods and ask questions in a calm environment. The arthropods chosen for handling (darkling beetles, Madagascar hissing cockroaches, and millipedes) were those that presented minimal risk of harm, ensuring a safe and positive experience for all participants.

Our post-session assessment revealed interesting insights about student preferences. While students could hold certain arthropods during the program, this did not necessarily determine which one they voted as their favorite at the end. For example, although the scorpion remained in its enclosure and was not handled by students or volunteers, it emerged as the overall favorite arthropod. This finding highlights that excitement about arthropods does not require physical interaction; observing them up close and seeing their unique characteristics (e.g., illuminating the scorpion under black light to demonstrate its fluorescent capabilities) can also generate significant interest. These results align with previous research showing that disgust reduction can occur even through non-hands-on experiences, such as studying photographs of animals [16]. In addition, previous research indicates that students with higher disgust sensitivity exhibit a greater preference for observational activities over hands-on activities [17]. Providing opportunities for observation, rather than exclusively hands-on experiences with arthropods, enabled these students to develop interest and have a positive experience, ultimately contributing to a reduction in disgust. Furthermore, bringing university experts to the elementary schools allowed students to learn from knowledgeable individuals who could convey confidence and comfort in the subject matter. This approach ensured that students were exposed to entomology in a positive light, which might not have been possible if their teacher harbored preconceived fears or negative attitudes toward arthropods [8,12].

Previous research suggests that out-of-school arthropod experiences can lead to a greater reduction in disgust compared to those held within elementary schools [7]. In addition, the novel experience of visiting a new environment to learn, known as the novel field trip phenomenon [18], can enhance students’ excitement and amplify their reactions. However, it is clear that exposure to arthropods organized and facilitated by experts effectively reduces students’ disgust, regardless of whether these experiences take place inside or outside of the school [7,19].

While these changes in learning environment can contribute to the success of elementary education, access to out-of-school programs is not always feasible. Factors like field trip expenses, difficulty securing parent volunteers, and logistical barriers (e.g., program closures during the COVID-19 pandemic) can pose challenges when planning off-site field trip events. Hokie BugFest on the Go addressed these issues by bringing arthropods and university experts directly to schools. This approach eliminated many of the common barriers associated with field trips while still providing an engaging small-group experience. Importantly, program leaders observed a significant reduction in students’ disgust toward arthropods, demonstrating the effectiveness of in-school programs when thoughtfully designed and facilitated by knowledgeable experts.

## 5. Conclusions

As educators and entomologists work to spread awareness and excitement about insects and other arthropods within their communities, understanding how to effectively engage specific groups is crucial. Our results demonstrate that bringing programs directly into schools is a successful way to reduce students’ disgust toward insects and other arthropods, particularly when local programs face challenges, such as field trip expenses or the inability to host larger, on-campus events. We recognize that our study addresses immediate effects and that further research is necessary to investigate the long-term impacts of this and similar programs on attitudes toward arthropods. These findings can guide organizers of similar entomology outreach events and inform long-term research efforts. By better understanding the comfort levels and preferences of young audiences, organizers can enhance the effectiveness and overall impact of their programs.

## Figures and Tables

**Table 1:** Voting box activity: comparison between pre- and post-session assessment responses (*n* = 713).

Question	Before Program	After Program			Significance Level
		Cute	Gross	Total	
Insects are?	Cute	487 (68.3)	5 (0.7)	492 (69.0)	<0.0001
	Gross	116 (16.3)	105 (14.7)	221 (31.0)	
	Total	603 (84.6)	110 (15.4)		
		Fun	Scary		
Insects are?	Fun	528 (74.1)	3 (0.4)	531 (74.5)	<0.0001
	Scary	101 (14.2)	81 (11.4)	182 (25.5)	
	Total	629 (88.2)	84 (11.8)		

Data expressed as number (%). Significance levels based on McNemar’s chi-square test.

**Table 2:** Group question/answer activity: comparison between pre- and post-session assessment responses (*n* = 713).

Question	Before Program	After Program			Significance Level
		Yes	No	Total	
Do you like insects?	Yes	615 (86.3)	0	615 (86.3)	<0.0001
	No	38 (5.3)	60 (8.4)	98 (13.7)	
	Total	653 (91.6)	60 (8.4)		
Do you think insects are important to humans?	Yes	668 (93.7)	0	668 (93.7)	<0.0001
	No	19 (2.7)	26 (3.6)	45 (6.3)	
	Total	687 (96.4)	26 (3.6)		
Will you touch or hold an insect during the program?	Yes	633 (88.8)	9 (1.3)	642 (90.0)	0.0061
	No	25 (3.5)	46 (6.5)	71 (10.0)	
	Total	658 (92.3)	55 (7.7)		

Data expressed as number (%). Significance levels based on McNemar’s chi-square test.

## Data Availability

The data presented in this study are available upon request from the corresponding author.

## Supplementary Materials

The following supporting information can be downloaded at https://www.mdpi.com/article/10.3390/insects16010093/s1, Handout S1: Hokie BugFest on the Go field trip activity booklet.
