# Supplementary material for: Transforming Children’s Attitudes Toward Insects Through In-School Encounters
Source: Insects. 2025 Jan 17;16(1):93. doi: 10.3390/insects16010093 (PMC11765737; doi:10.3390/insects16010093)
Supplement: Supplementary file 1 [file insects-16-00093-s001.zip › insects-3403268-supplementary.pdf]

2022 Traveling Field Trip

Hokie BugFest

COLLEGE OF AGRICULTURE AND LIFE SCIENCES
ENTOMOLOGY
VIRGINIA TECH

VTPP
Virginia Tech Pesticide Programs

Virginia Cooperative Extension
Virginia Tech • Virginia State University

www.ext.vt.edu

Hokie BugFest

T	R	E	H	H	I	N	S	E	C	T	O	S	M
N	E	C	G	B	N	E	M	O	D	B	A	G	I
D	D	T	A	L	U	T	N	A	R	A	T	N	L
H	I	E	V	R	E	T	I	C	F	T	I	I	L
E	P	I	R	S	T	C	T	V	V	N	X	W	I
A	S	K	A	H	H	H	A	E	M	A	C	T	P
D	T	O	L	T	D	A	R	N	R	R	A	A	E
B	R	H	O	B	I	N	M	O	I	F	D	R	D
U	B	M	L	I	R	C	H	C	P	M	L	A	E
G	E	E	R	S	U	T	K	O	E	O	A	Y	T
F	E	E	A	N	N	E	T	N	A	E	D	L	F
E	T	N	E	N	T	O	M	O	L	O	G	Y	A
S	L	B	C	H	A	S	S	C	I	E	N	C	E
T	E	S	D	R	A	G	O	N	F	L	Y	N	T

Antennae
Beetle
Butterfly
Hokie
Science
Millipede

Dragonfly
Moth
Head
Larva
Insect
Animal

Entomology
BugFest
Abdomen
Arthropod
Spider
Tarantula

Wings
Thorax
Cricket
Tick

My Hokie Bugfest Field Trip

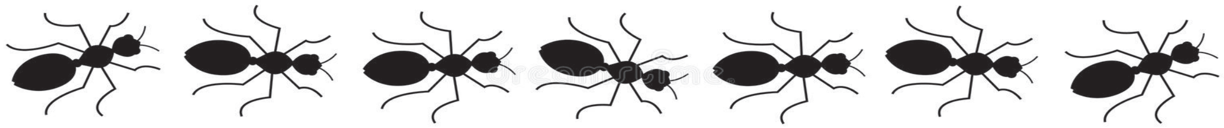

The bug I remember MOST at the field trip was the ____________________.

The most interesting thing about it was ________________________________.

CIRCLE all the bugs you remember seeing at the field trip

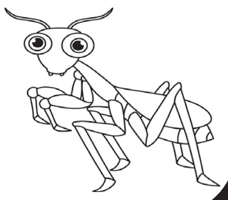

Millipede Tarantula Cockroach Scorpion Termite

Blue Death Feigning Beetle Praying Mantid

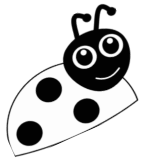

I **TOUCHED** a bug during the field trip: YES NO

The bug I **TOUCHED** was a ______________________.

It felt _______________________________________.

I did **NOT** touch a bug because ______________________________________.

I **HELD** a bug during the field trip: YES NO

The bug I **HELD** was a __________________________.

It felt _______________________________________.

I did **NOT** hold a bug because _______________________________________.

One thing I learned at the field trip that I did NOT already know is

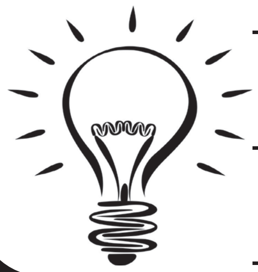

____

____

____.

If I was a bug, I would want to be a _______________.
It would be cool to be this kind of bug because _____________
____.

My Feelings About Bugs

I think bugs are...

(Circle all that apply)

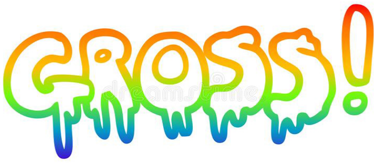

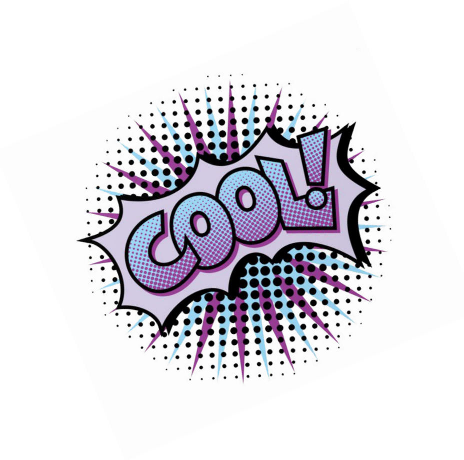

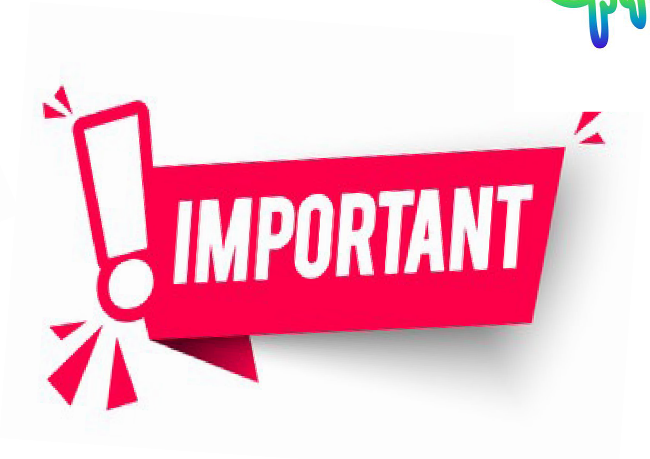

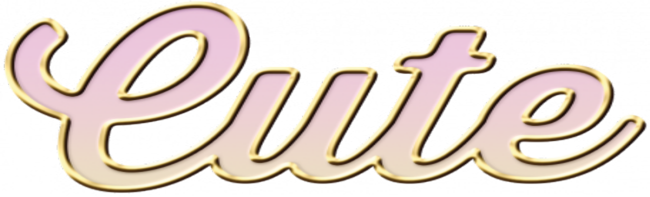

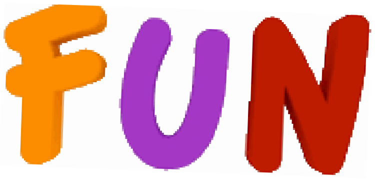

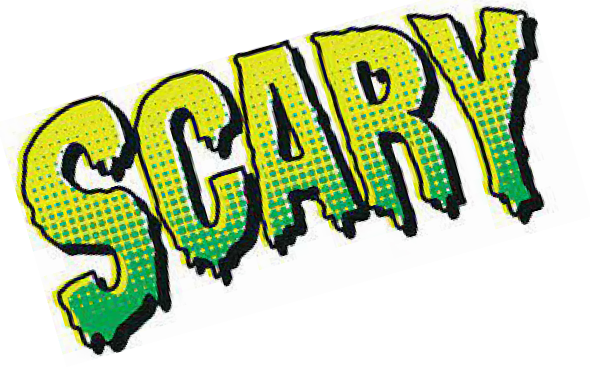

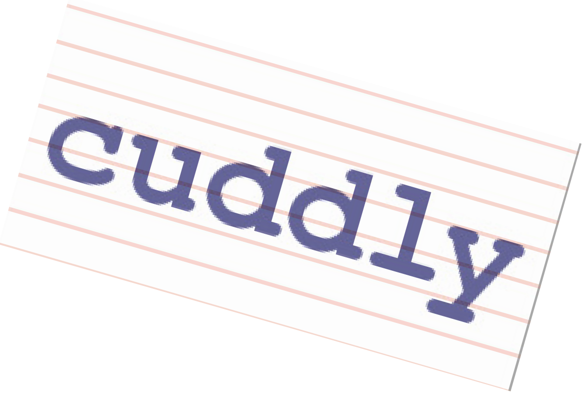

Create an acrostic poem using insect related words!

E

N

T

O

M

O

L

O

G

Y

Label the Insect

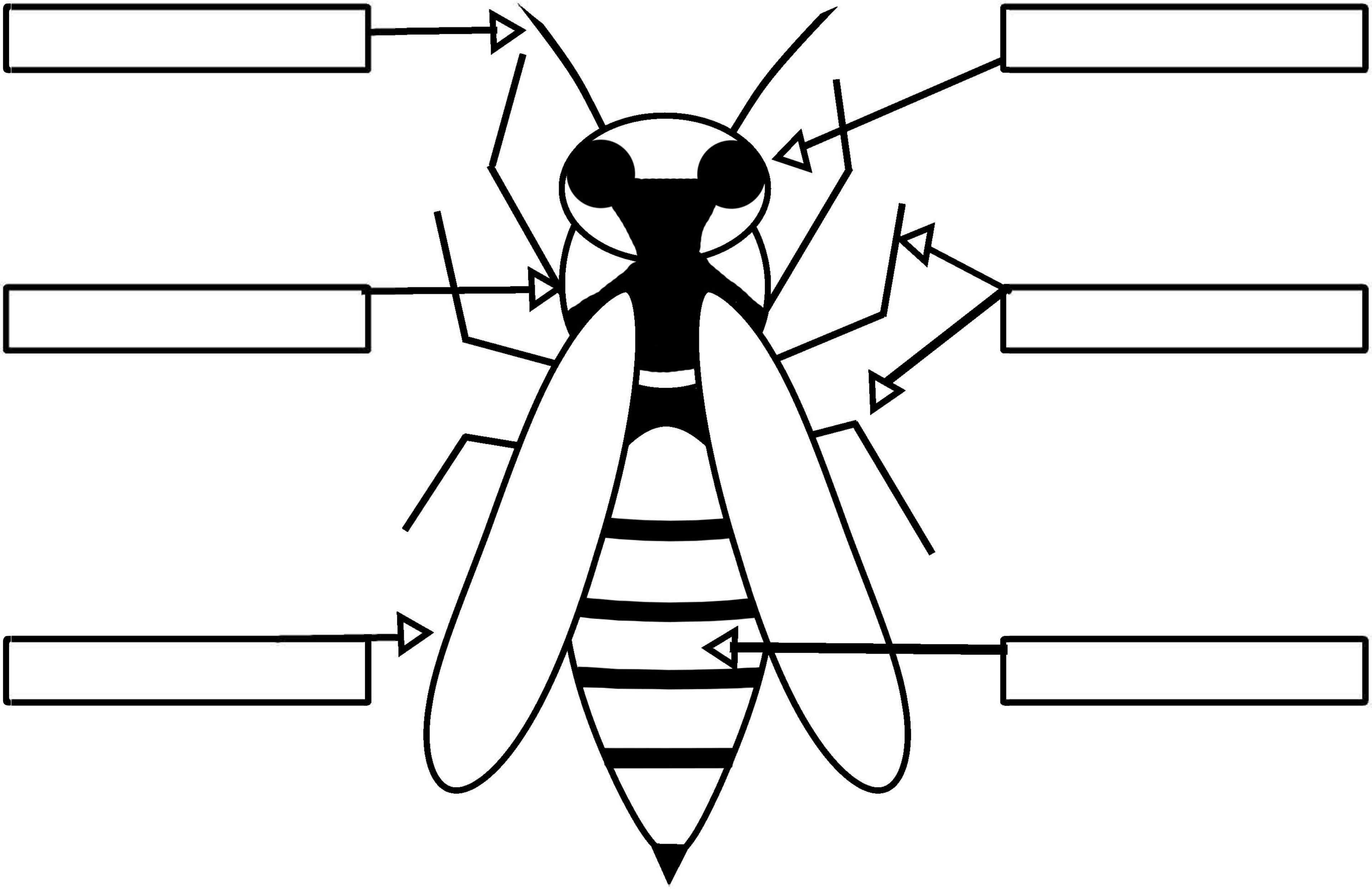

Antenna Abdomen Head Legs Thorax Wings

Complete Metamorphosis

Put the steps of metamorphosis in order by drawing a line to match each picture to the numbers 1-4.

Pupa

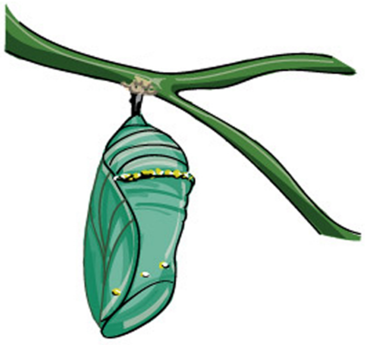

1

Adult

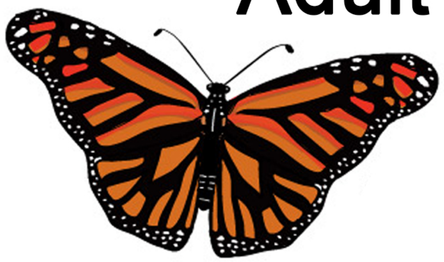

2

3

Egg

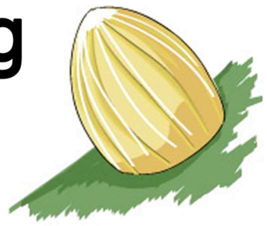

Larva

4

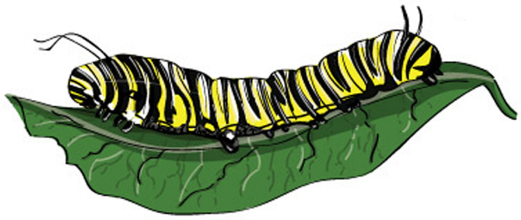

TRUE OR FALSE
Circle what you think is true!

Earthworms are insects.

Insects have 3 body segments.

Bugs don’t benefit humans.

Insects are arthropods.

Ticks and spiders are arthropods.

Insects were present on Earth before the dinosaurs.

Insects have 2 pairs of antennae.
